# Characterization of Upper Limb Impairments at Body Function, Activity, and Participation in Persons With Multiple Sclerosis by Behavioral and EMG Assessment: A Cross-Sectional Study

**DOI:** 10.3389/fneur.2019.01395

**Published:** 2020-02-14

**Authors:** Nicola Valè, Marialuisa Gandolfi, Stefano Mazzoleni, Elena Battini, Eleonora Kirilova Dimitrova, Alberto Gajofatto, Francesco Ferraro, Matteo Castelli, Maruo Camin, Mirko Filippetti, Carola De Paoli, Alessandro Picelli, Jessica Corradi, Elena Chemello, Andreas Waldner, Leopold Saltuari, Nicola Smania

**Affiliations:** ^1^Department of Neurosciences, Biomedicine and Movement Sciences, University of Verona, Verona, Italy; ^2^UOC Neurorehabilitation, AOUI Verona, Verona, Italy; ^3^The BioRobotics Institute, Scuola Superiore Sant' Anna, Pontedera, Italy; ^4^UOC Neurologia dU, Azienda Ospedaliera Universitaria Integrata, Verona, Italy; ^5^Section of Neuromotor Rehabilitation, Department of Neuroscience, ASST Carlo Poma, Mantova, Italy; ^6^Centro di Riabilitazione Franca Martini–ATSM ONLUS, Trento, Italy; ^7^School of Specialization in Physical Medicine and Rehabilitation, University of Verona, Verona, Italy; ^8^Department of Neurological Rehabilitation, Private Hospital Villa Melitta, Bolzano, Italy; ^9^Research Department for Neurorehabilitation South Tyrol, Bolzano, Italy; ^10^Department of Neurology, Hochzirl Hospital, Zirl, Austria

**Keywords:** upper limb abnormalities, quality of life, participation, electromyography, multiple sclerosis

## Abstract

**Background:** Multiple sclerosis (MS) is a chronic inflammatory demyelinating and disabling disease which primarily affects individuals in their early life between 20 and 40 years of age. MS is a complex condition, which may lead to a variety of upper limb (UL) dysfunctions and functional deficits.

**Objective:** To explore upper limb impairments at body function, activity, and participation in persons with MS (PwMS) and severe hand dexterity impairment by behavioral and surface electromyography (sEMG) assessments.

**Methods:** This observational cross-sectional study involved 41 PwMS with severe hand dexterity impairment stratified according to the Expanded Disability Status Scale (EDSS) into mild–moderate (*n* = 17; EDSS, 1–5.5), severe ambulant (*n* = 15; EDSS, 6–6.5), and severe nonambulant (*n* = 9; EDSS, 7–9.5). Behavioral outcome measures exploring body function, activity, and participation were administered. The sEMG activity of six upper limb muscles of the most affected side was measured during a reaching task.

**Results:** The most severe group was significantly older and more affected by secondary progressive MS than the other two groups. Positive significant associations between UL deterioration and impairments at different International Classification of Functioning, Disability, and Health domains were noted in the most severe group. The progressive decline in manual dexterity was moderately to strongly associated with the deterioration of the overall UL activity (ρ = 0.72; *p* < 0.001) and disuse (amount of use ρ = 0.71; *p* < 0.001; quality of movement ρ = 0.77; *p* < 0.001). There was a low correlation between manual dexterity and UL function (ρ = 0.33; *p* = 0.03). The muscle activation pattern investigated by sEMG was characterized by a decrease in modularity and timing delay in the wrist extensor muscles activation in the severe ambulant patients (EDSS, 6–6.5). Similar impairments were observed in the proximal muscles (anterior deltoid) in the more advanced stages (EDSS ≥ 7).

**Conclusion:** Behavioral assessment, together with measures of muscle activation patterns, allows investigating the pathophysiology of UL impairments in PwMS across progressive neurological disability severity to implement task-specific rehabilitation interventions.

## Introduction

Multiple sclerosis (MS) is the most common non-traumatic cause of neurological disability in young adults between 20 and 40 years old, affecting about 2–3 million people globally (1, 2). A greater understanding of the underlying genetic and environmental factors involved in the MS pathophysiology has been reached, followed by early accurate diagnosis and extensive therapeutic management toward more personalized medicine (2). However, MS continues to be a challenging condition both in the treatment and prevention of the disabling progression of the disease, especially in the progressive forms (2).

Rehabilitation plays an integral part in the management of people with multiple sclerosis (PwMS). In the last decade, special attention has been paid on gait and balance disturbances, as they have been considered to be one of the key determinants of mobility limitations and disability (3). However, up to 66% of these patients suffer from upper limb (UL) dysfunctions (4). Since MS typically affects multiple functional systems, a variety of symptoms significantly impact on the patient's ability to perform activities of daily living and quality of life (QoL) (4). Common manifestations include muscle weakness, tremor, sensory deficits, and impaired motor control (2). Fatigue and pain further impair motor and functional outcomes (2).

The pathophysiology of UL impairments in PwMS is complex and only partially known. The bulk of the literature on this topic suggests that sensorimotor dysfunctions are not the only mechanism to explain UL disability (5). For instance, cognitive impairments (i.e., attentional and memory deficits) and UL disuse might further affect UL function with negative consequences on activity and participation (6–11).

Two issues need to be addressed to improve knowledge on the pathophysiology of UL impairments. First, specific UL assessment protocols should be shared among clinicians and researchers. Second, technology-aided assessments should be integrated into the UL assessment to explore function from a qualitative and quantitative point of view. An accurate UL assessment is challenging due to the inherent variability of UL movements and the multifaceted manifestation of UL dysfunctions in PwMS. Of note, the standard neurological disability assessment using the Expanded Disability Status Scale (EDSS) is mainly focused on mobility and walking ability (8). As highlighted in the recent overview by Lamers et al. (8), UL dysfunction in PwMS should be investigated within the International Classification of Functioning, Disability, and Health (ICF) framework including outcome measures referring to body structure and function, activity, and participation (8). A consensus of diagnostic hand dexterity impairment criteria using the Nine-Hole Peg Test (NHPT) has been reached. These criteria should improve the accuracy of epidemiological studies and allow to monitor sensorimotor function in PwMS (3, 12, 13). Of note, a specific altered pattern of hand movement can reflect brain maladaptation (14). However, the relationship between different levels of UL impairments deserves further investigation (5, 12, 15).

The literature on the instrumental assessment of UL dysfunctions in PwMS is scant. Constraints in the use of such technology are the costs to acquire the technological devices, the need for specific expertise to acquire and analyze data, and the time requested to set up the instrumental paradigm. Strengths are the possibility to use it during functional tasks and activity. Scattered evidence in PwMS suggests that surface electromyography (sEMG) has many advantages over other neurophysiological investigations to study muscle activation pattern including non-invasive assessment, the possibility to describe muscle activation during a controlled and repeatable functional task, and the affordable costs in the rehabilitation setting. Preliminary studies on small samples have reported lower modulation in sEMG activity of distal UL muscles in patients with moderate impairment during reaching to grasp task (16). A more recent study by Pellegrino et al. (17) suggested that both kinematic and electromyographic parameters might represent biomarkers to help clinicians in differentiating patients with different levels of UL motor impairment from healthy subjects (17). Noteworthy, no patients with severe UL impairments have been investigated, and no correlation between clinical outcome and sEMG data was performed.

To accomplish this goal, we explored UL impairments at different levels of ICF by behavioral and sEMG assessment in a cohort of PwMS affected by severe hand dexterity impairment and different levels of neurological disability.

Knowledge gained from this study will provide new insights into the progressive deterioration of UL function and activity across the different disease stages, as a thorough investigation of the UL impairments at different ICF domains would show that manual dexterity deficit may be associated with multiple UL dysfunctions depending on the neurological disability. Preliminary analysis of UL muscle activation may suggest changing in the modularity and timing of UL muscle activation during a reaching task as sEMG correlates of the UL decline in PwMS (16). Our preliminary results would be a reference for prospective longitudinal studies on a large cohort of patients to study behavioral and muscle activation pattern deterioration in the different stages of the illness identified by the EDSS.

## Methods

In this observational cross-sectional study, we used data provided by a database created for a randomized controlled trial on hand dexterity robot-assisted rehabilitation by Gandolfi et al. (18). A total of 113 patients were screened at the UOC Neurorehabilitation Unit (AOUI Verona) and the Multiple Sclerosis Center, U.O. Neurologia dU (AUOI Verona) from March 2014 to March 2017. Inclusion criteria were the following: confirmed diagnosis of MS (19); age between 18 and 65 years, EDSS score 1.5 < *x* < 8 (19); Mini-Mental State Evaluation ≥ 24/30 (20); Modified Ashworth Scale score evaluated at the elbow, wrist, and fingers ≤2 (21); and NHPT score between 30 and 300 s (13). Exclusion criteria were the absence of relapses or relapse-related treatments in the 3 months before the study, and other neurological or orthopedic diseases interfering with UL function. After being informed about the experimental nature of the study, patients gave their informed written consent. The study was carried out following the Helsinki Declaration, approved by the local Ethics Committee (prog no. 230 CESC), and registered at a clinical trial. Eligible patients were categorized into three disability groups according to the EDSS: group 1, mild–moderate (1.5–5.5); group 2, severe ambulant (6–6.5); and group 3, severe non-ambulant (5). The EDSS was used to categorize the different disease stage severity, as it is the worldwide measure of neurological impairment in PwMS already used in the literature (5).

## Assessments

A neurologist determined demographic and clinical data such as gender, age, hand dominance determined by the Edinburg Handedness Inventory (EHI) (22), disease duration (years), type of MS, and the EDSS. According to the ICF, clinical and instrumental assessments were administered by a research therapist to explore body function, activity, and participation. The ICF is the WHO framework for measuring health and disability at the individual and population levels, taking into account environmental and personal factors (23). The body function domain refers to the physiological function of the body system, including psychological functions. The activity domain refers to the execution of a task or action by the individual. The participation level describes the personal involvement in real-life situations (23). Clinical assessments were carried out by blinded assessors about the EDSS score cut points defining the different study groups. A physiotherapist with experience in the sEMG acquisition acquired instrumental assessments. Data processing was carried out by external research collaborators not involved in the data collection to limit possible performance and detection bias.

### Upper Limb Functions and Structures

The Fugl–Meyer–UL section (FM) was used as a measure of UL function. FM includes evaluation of reflex activity, volitional movement, and coordination of the UL (range of score, 0–66; higher = better performance) (24). The Motricity Index measured muscle strength at the shoulder, elbow, and pinch grip (range of score, 0–100; higher = better performance) (25). The Modified Ashworth Scale (MAS) measured resistance during passive stretching of shoulder adductors, elbow, and wrist flexors (range of score, 0–5; higher = worse performance) (21). A total UL MAS score was also computed as the sum of shoulder abductors, elbow, and wrist flexors single scores (21). Tremor Severity Scales assessed the UL tremor across four domains: rest tremor, postural tremor, kinetic tremor, and intention tremor (range of score for each domain, 0–10; higher = more severe tremor) (26). Numeric Rating Scale assessed fatigue. Patients answered the question “Do you perceive fatigue during UL activity?” (range of score, 0–10; higher = worse symptoms) (27).

### Upper Limb Activity

The NHPT was used to evaluate manual dexterity by computing the pegs *per second* (peg/s) rate. The NHPT has been reported to be responsive to UL activity level worsening (12, 13, 28). A previous study by Lamers et al. (15) suggested that the scores below 0.27 pegs/s indicate a severe hand dexterity deficit (15). The action research arm test (ARAT) measured functional arm skills with 19 items categorized in four sections (grasp, grip, pinch, and gross) (range of score, 0–57; higher = better performance) (29). The motor activity log (MAL) assessed changes in the amount and the quality of the arm use in accomplishing 30 daily activities (range of score, 0–168; higher = better performance) (30).

### Participation Level

The Multiple Sclerosis Quality of Life-54 with the physical health and mental health domains measured generic and MS-specific domains of health-related QoL (range of score, 0–100; higher = better performance) (31). The patient's satisfaction with daily activities or social roles was assessed using the Life Habits Assessment–general short form (32).

### Instrumental Assessment

**Figure 1:**
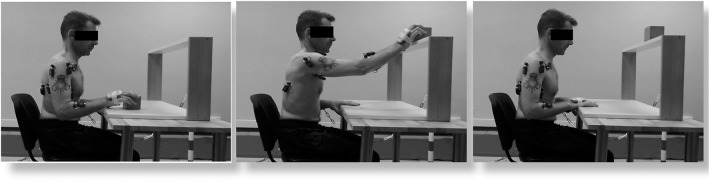
The reaching task (ARAT grasp subscale). The movement was divided into three phases, as shown.

The patients underwent one session (three trials/session) of sEMG acquisition as follows. The sEMG amplitude domain from six upper limb muscles of the more affected side (deltoid scapular, deltoid clavicular, triceps brachii, biceps brachii, flexor carpi radialis, and extensor carpi radialis) was measured using pairs of self-adhesive surface electrodes. The sEMG signals from trapezius inferior and pectoralis major along with data from the inertial sensor fixed to the subjects' hand were not considered because of the low quality of the signals acquired. Disposable Ag–AgCl electrodes were placed according to the Surface Electromyography for the Non-invasive Assessment of Muscles guidelines with an interelectrode spacing of 0.02 m. Before electrode placement, the skin was shaved with a disposable, single-use razor and cleaned with alcohol (33). Raw sEMG signals were collected using BTS FREEEMG 300 wireless sEMG sensors (BTS spa, Milan, Italy) at a sampling rate of 1,000 Hz. Raw sEMG signals were processed with a customized routine developed in MATLAB environment (MathWorks, USA). The raw sEMG signal was bandpass filtered at 20–450 Hz and then smoothed using a 20-ms root mean square algorithm to obtain the envelope. Signals were recorded during a functional task of reaching while grasping an object (ARAT grasp section; Figure 1). Patients sat upright in a standard chair with a firm back without armrests according to the ARAT standard procedures (34). Patients were asked to keep the trunk in contact with the back of the chair during testing. The UL was positioned in pronated position on the table. The task consisted of grasping and placing a 7.5-cm wooden cube on a shelf of standardized height (37 cm) corresponding to the grasp item of the ARAT testing. The task was divided into three phases by identifying four temporal events (start, grasping the cube, placing the cube on the shelf, returning to initial position). The task was repeated three times with a resting time of 2 min between trials, and the signals were averaged and time normalized. Normative data were collected on a convenient sample involving 10 healthy age-matched controls undergoing one session (three trials/session) of the same sEMG acquisition protocol. The sEMG paradigm is illustrated in Figure 1.

## Data Analysis

Results about the most affected UL were reported. Descriptive statistics included median and first through third quartiles (Q1; Q3) to describe the magnitude of UL impairments on the different ICF domains in the whole group and the different EDSS subgroups. Since the data were not normally distributed (Shapiro–Wilk test), non-parametric tests were used for inferential statistics. The Kruskal–Wallis H test (“one-way ANOVA on ranks”) was used to determine statistically significant differences between the three groups of the independent variables. *Post hoc* between-group comparisons were performed using the Mann–Whitney *U* test (corrected for multiple comparisons using Tukey's multiple comparisons test).

As manual dexterity was previously showed to play a key role in the UL overall impairment, linear correlations between the NHPT and other outcome measures were computed using Spearman's correlation in the all sample to explore the strength of the relationship among outcome measures. Data distribution did not allow to perform a linear regression model, and the correlation strength was defined as very high (ρ > 0.9), high (ρ = 0.7–0.89), moderate (ρ = 0.5–0.69), low (ρ = 0.3–0.49), or very low (ρ < 0.29) (35).

The sEMG data were qualitatively and quantitatively analyzed. The normalized mean sEMG envelope for healthy subjects and each patients' subgroup was used to display the muscle activation patterns during the movement and reported in figures. Moreover, the timing of maximal muscle activation for each phase was calculated as a percentage of the relative movement phase (1–100%) for each group. One-way ANOVA was used to determine statistically significant differences among groups. *Post hoc* between-group comparisons were performed using the Mann–Whitney *U*-test. According to the functional involvement of the six muscles during the reaching task, muscles were coupled as follows: (1) deltoid clavicular and biceps brachii, as shoulder flexors; (2) biceps and triceps brachii, as agonist and antagonist actors during the elbow flexion; and (3) flexor and extensor carpi radialis because involved in maintaining the wrist in neutral position during the wooden cube displacement. The mean difference between the timing of maximal muscle activation for each couple of muscles in each subgroup of PwMS patients was computed. No frequency domain data analysis was performed. Statistical analysis was carried out with SPSS 20.0 (IBM SPSS Statistics for Windows, Version 20.0, Armonk, NY, USA) and Stata/IC 15.1 for MAC (StataCorp, TX, USA).

## Results

Forty-one patients have been included and allocated to mild–moderate (*n* = 17), severe ambulant (*n* = 15), and severe non-ambulant (*n* = 9) groups. All patients were assisted by family members and were living in their home. No patients were institutionalized or community-dwelling PwMS. Significant between-group differences in age (*p* = 0.05) and the type of MS among groups were measured. The most severe group was older than the other two groups. Moreover, the majority of patients in the most severe group were affected by secondary progressive MS. Table 1 reports demographic and clinical characteristics of the sample.

**Table 1:** Demographic and clinical characteristic of the sample.

		**MS subgroups**			***p*-value**
		**Group 1**	**Group 2**	**Group 3**	
	**Total (*n* = 41)**	**Mild–moderate EDSS (1–5.5) (*n* = 17)**	**Severe ambulant EDSS (6–6.5) (*n* = 15)**	**Severe NA EDSS (7–9.5) (*n* = 9)**	
Gender (F/M)	25/16	12/5	7/8	6/3	0.35
Age (years)	50.88 ± 10.9	45.88 ± 11.98	54.07 ± 9.16	55 ± 8.35	0.05^*^
EDSS	6 (4.25–6.5)	4 (3.5–5.25)	6 (6–6.5)	7.5 (7-8)	<0.001^*^
EDSS sensory	2 (0.5–2)	1 (0–2)	2 (2)	1.5 (0.5–2)	
Hand dominance (R/L/A)	36/5/0	13/4/0	14/1/0	9/0/0	0.16
Disease duration (years)	14.20 ± 8.76	12.12 ± 9.5	14.60 ± 8.52	17.44 ± 6.23	0.29
Type of MS (PP/RP/RR/SP)	2/2/22/15	0/1/13/3	1/1/8/5	1/0/1/7/9	0.05^*^
Visual impairment (yes/no)	2/39	0/17	1/14	1/8	0.70

*Data are presented as frequency mean ± SD or median (25th/75th percentiles)*.

*F, female; M, male; EDSS, Expanded Disability Status Scale; NA, nonambulant; R, right; L, left; A, ambidextrous; MS, Multiple sclerosis; RR, relapsing remitting; SP, secondary progressive; PP, primary progressive*.

* *Significant p-value*.

### Body Functions and Structures Level

A statistically significant difference was found in UL function (FM scale), and muscle tone (MAS), and the fatigue perceived were measured (Table 2) among groups. *Post hoc* comparisons showed that participant in the severe non-ambulant group (group 3) experienced significantly higher UL muscle tone than the mild–moderate group (group 1) (*p* = 0.002). Fatigue was significantly higher in the severe ambulant group (group 2) than the mild–moderate group (group 1) (*p* = 0.008) and significantly lower in the severe nonambulant group (group 3) than the severe ambulant (*p* = 0.004).

**Table 2:** Median scores (25th /75th percentiles) of clinical variables and *p* values comparing the three groups.

		**MS subgroups**			
		**Group 1**	**Group 2**	**Group 3**	
	**Total (*n* = 41)**	**Mild–moderate EDSS (1–5.5) (*n* = 17)**	**Severe ambulant EDSS (6–6.5) (*n* = 15)**	**Severe NA EDSS (7–9.5) (*n* = 9)**	***p* value**
**Body function and structures**					
Fugl–Meyer (0–66)	60 (43–64.5)	63 (56.5–65)	56 (42–64)	48 (36.5–61)	0.037^*^
Motricity Index (0–100)	85 (73–93)	92 (74–93.75)	83 (71–94.75)	73 (64.5–90.5)	0.179
- Pinch grip (0–33)	26 (22–33)	26 (26–33)	26 (24–29.5)	22 (22–26)	0.169
- Elbow flexor (0–33)	25 (25–33)	33 (25–33)	25 (22–33)	25 (25–33)	0.579
- Shoulder abductors (0–33)	25 (25–33)	33 (25–33)	25 (25–33)	25 (19–33)	0.125
Modified Ashworth Scale (0–15)	0 (0–1)	0 (0–0.5)	0 (0–2)	2 (0.5–3)	0.008^*^
- Elbow flexors (0–5)	0 (0–1)	0 (0–0)	0 (0–0.5)	1 (0–1)	0.004^*^
- Wrist flexors (0–5)	0 (0–0)	0 (0–0)	0 (0–0)	0 (0–0.5)	0.056
- Finger flexors (0–5)	0 (0–0)	0 (0–0)	0 (0–0.5)	0 (0–1)	0.395
Tremor Severity scale (0–5)	0 (0–2)	0 (0–2)	0 (0–3)	0 (0–2.5)	0.689
Numeric Rating Scale fatigue	6 (5–8)	6 (5–7.5)	8 (7–9)	5 (5–7.5)	0.005^*^
**Activity level**					
NHPT (pegs/sec)	0.23 (0.14–0.27)	0.26 (0.23–0.28)	0.18 (0.15–0.26)	0.07 (0.04–0.20)	0.027^*^
ARAT (0–57)	49 (39.59–53)	52 (48–53)	47 (34–52)	40 (16.5–52.5)	0.145
- Grasp (0–18)	18 (12–18)	18 (18)	15.5 (11.75–18)	13 (6–18)	0.054
- Grip (0–12)	10 (8–12)	12 (11–12)	10 (7.75–12)	8 (6–10.25)	0.01^*^
- Pinch (0–18)	13 (12–17.5)	15 (12.25–15.75)	13 (9.75–18)	12 (2–13)	0.660
- Gross (0–9)	9 (7–9)	9 (9)	9 (5.75–9)	8 (4–9)	0.052
MAL AoU	113.5 (76.25–132.75)	131 (115.5–147.5)	113 (92–132)	69.5 (27.25–82.75)	0.005^*^
MAL QoM	108.75 (86.125–126.75)	123 (110.5–130.5)	108.5 (100–119)	69 (24–80.5)	0.001^*^
**Participation level**					
LifeH	11.85 (9.97–12.91)	12.7 (12.1–14.53)	11.56 (8.95–12.1)	10.4 (8.72–10.66)	<0.001^*^
MSQOL-54	102.17 (60.8–129.84)	127.93 (60.22–146.15)	65.64 (51.2–110.49)	103 (78.57–121.32)	0.160

*Data are presented as median (25th/75th percentiles)*.

*EDSS, Expanded Disability Status Scale; MS, multiple sclerosis; NA, non-ambulant; NHPT, Nine-Hole Peg Test; ARAT, action research arm test; MAL, motor activity log; MSQoL-54, Multiple Sclerosis Quality of Life-54; LifeH, Life Habits assessment–general short form*.

* *Significant p-value*.

### Activity Level

**Figure 2:**
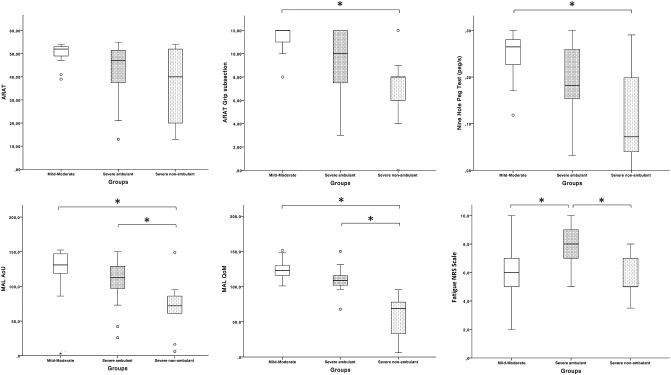
Median scores of clinical variables comparing the three subgroups. ^*^Significant *p*-value.

Manual dexterity assessed with the NHPT differed significantly among groups (Table 2). PwMS showed a progressive decrease of peg/second rate and a progressive reduction in UL use according to the neurological disability (Table 2). The severe non-ambulant participants (group 3) experienced significantly higher impairments than the mild–moderate group (group 1) in the NHPT (*p* = 0.015) and MAL (*p* < 0.001). The NHPT score was strongly correlated to ARAT (ρ = 0.721, *p* < 0.001), MAL-AOM (ρ = 0.712, *p* < 0.001), and MAL-QOM (ρ = 0.769, *p* < 0.001). Conversely, there was a low correlation between FM and NHPT score. Within the ICF framework, outcome measures belonging to the activity domain were moderate to very strongly correlated with each other. The MAL score in the severe ambulant participants (group 2) differed significantly from the severe non-ambulant group (group 3) (*p* = 0.001). No significant differences in the ARAT total score were measured. However, the grip subsection score was statistically different among groups (*p* = 0.01). *Post hoc* comparisons showed that the severe non-ambulant group (group 3) performance was worse than the mild–moderate group (group 1) (Table 2, Figure 2).

### Participation Level

The patient's satisfaction with daily activities or social roles assessed by the Life Habits Assessment–general short form differed significantly among groups (Table 2). Participants in the mild–moderate group (group 1) reported significantly higher satisfaction than the severe ambulant (group 2) (*p* = 0.001) and severe non-ambulant (group 3) group (*p* < 0.001). *Post hoc* comparisons are shown in Figure 2.

### Instrumental Assessment Results

**Figure 3:**
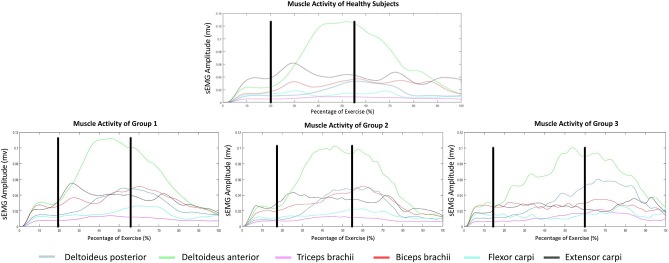
Mean surface sEMG envelope of healthy subjects **(Top)** and patients **(Bottom)**.

The mean sEMG envelope in healthy controls showed a relevant activation of deltoid anterior between phases 2 and 3, corresponding to the deltoid's typical recruitment during the shoulder flexion. The triceps brachii activity was almost absent during the entire movement, while a slight activation of the biceps brachii was recorded during phases 2 and 3. Although the overall activation value for flexor carpi muscles was <0.02 mV, a modulation in its activity, associated with the activity of higher intensity of extensor carpi activation, was found at about 30% and 70–80% of the movement. This coupled activation was consistent with forearm muscles' function of wrist stabilization during a grasping task. In PwMS, a progressive decrease in the modularity of muscle activation was reported in association with increasing in neurological disability. This loss of modularity was especially evident in the flexor and extensor carpi muscles, where the most severe patients showed a constant (but low) muscle activation (Figure 3).

The between-group analysis of the timing of maximal muscle activation showed statistically significant differences in the anterior deltoid in phase 3 (*p* = 0.034), which was during the eccentric contraction of the muscle, and in the extensor carpi muscles during phase 2 (*p* = 0.020), while subjects were holding the cube (Table 3). The *post hoc* analysis showed that severe non-ambulant patients reported a delayed maximal activation of the deltoid anterior compared to other groups in the last movement phase (*p* = 0.027; adjusted for multiple comparisons). Similarly, extensor carpi muscles were activated with a significant delay in the severe group compared with the mild–moderate patients in phase 2 (*p* = 0.043; adjusted for multiple comparisons). Interestingly, the difference of the maximal activation of extensor carpi muscles between patients and healthy subjects during the holding-cube phase was found to be significantly correlated to the performance at the NHPT (ρ = −0.44; *p* = 0.038).

**Table 3:** Mean (standard deviation) of timing maximal muscle activation.

	**Movement phase**	**Healthy subjects**	**MS subgroups**			***p*-value**
			**Group 1**	**Group 2**	**Group 3**	
			**Mild–moderate**	**Severe ambulant**	**Severe NA**	
			**EDSS (1–5.5)**	**EDSS (6–6.5)**	**EDSS (7–9.5)**	
Anterior deltoid	1	72.5 (25.6)	71.3 (23.4)	84.6 (15.2)	65.5 (24.3)	0.58
	2	81.4 (15.0)	66.5 (17.9)	78.6 (18.7)	82.8 (6.9)	0.14
	3	2.7 (3.8)	6.2 (7.2)	5.8 (11.6)	23.5 (27.4)	0.034^*^
Biceps brachii	1	65.1 (22.5)	57.2 (22.6)	59.4 (25.4)	64.5 (24.0)	0.86
	2	67.5 (31.2)	56.17 (38.0)	67.8 (29.9)	59.3 (28.9)	0.84
	3	42.9 (29.5)	32.8 (22.6)	39.6 (20.2)	30.0 (32.7)	0.75
Triceps brachii	1	67.1 (17.3)	73.4 (20.0)	68.0 (21.9)	54.0 (30.2)	0.47
	2	68.5 (9.5)	68.6 (26.5)	48.8 (35.2)	84.8 (15.6)	0.14
	3	15.1 (13.3)	18.7 (26.6)	27.2 (32.8)	25.0 (25.7)	0.77
Flexor carpi	1	82.7 (15.8)	74.5 (21.4)	60.8 (24.7)	63.8 (16.0)	0.16
	2	34.4 (34.9)	70.6 (37.3)	47.0 (43.7)	50.0 (38.5)	0.19
	3	22.0 (13.3)	32.2 (31.7)	59.2 (45.3)	64.0 (45.6)	0.07
Extensor carpi	1	81.1 (21.6)	73.6 (29.3)	52.0 (23.0)	65.0 (23.6)	0.18
	2	27.2 (15.3)	26.3 (24.6)	65.4 (34.8)	54.5 (43.2)	0.020^*^
	3	41.5 (31.9)	29.0 (28.0)	15.4 (15.3)	50.3 (30.2)	0.19

*Data are reported as movement phase percentage*.

* *Significant p-value*.

**Figure 4:**
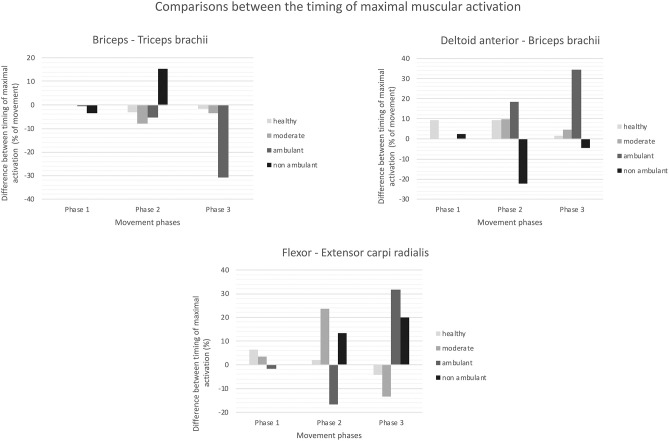
Difference between the timing of maximal activation for functionally coupled muscles.

The comparison between the difference in the timing of maximal activation between functionally coupled muscles did not show any significant difference between groups. However, during phase 2, the maximum of proximal muscles activation (biceps brachii and deltoid anterior) occurred almost simultaneously in healthy controls and moderate and severe ambulant patients (groups 1 and 2). Conversely, severe non-ambulant patients (group 3) showed a remarkable decrease in the modularity of biceps brachii, which resulted in a different sequence in the timing of activation. Similarly, the difference in temporal activation of the biceps and triceps brachii showed an opposite behavior between the severe non-ambulant group and the other two groups. More in detail, patients of group 3 activated the triceps brachii more than other patients, and the lack of modularity of biceps activity prevents the calculation of the maximum of the envelope. Concerning the forearm muscles, the differences between the timing of maximal activation between the flexors and extensor muscles, both in phases 2 and 3, were remarkably higher in all groups as compared to the healthy controls (Figure 4).

## Discussion

The results of this observational cross-sectional study suggest that manual dexterity might be already impaired in the mild–moderate stages when the overall neurological disability is low. Strong to moderate significant associations between UL deterioration and impairments at different ICF levels were noted in the most severe group. The progressive decline in manual dexterity, as measured by the NHPT, was associated with the deterioration of the overall UL activity (measured by ARAT) and disuse. Weakness, sensory loss, and tremor seemed not to be significant determinants of UL deterioration in our cohort. The sEMG assessment showed impairments in both modularity and timing of activation of distal (extensor carpi) muscles in the severe ambulant patients, while impairments in the timing of activation in the proximal muscles (anterior deltoid) were found in the more advanced stages (EDSS ≥ 7).

To the best of our knowledge, this study is the first one exploring objectively and subjectively UL dysfunction at different ICF levels using clinical and instrumental assessments. Strength in the methodology includes the fact that all patients suffered from severe manual dexterity impairment and that clinical and instrumental assessments were combined to explore the multifaceted nature of UL dysfunction. Limitations are the small sample of patients with severe neurological impairments and the lack of prospective longitudinal assessments. Concerning previous literature, our data confirm positive associations between manual dexterity and UL function and activity level and further explore new associations for discussion.

According to the literature, manual dexterity impairment is one of the major determinants of disability in PwMS since the first stages of the illness (5, 15). Hand dexterity plays a crucial role in everyday life activities and social participation, as it has been reported to be associated with independence in activities of daily living and UL use (36). The NHPT is recommended as a gold standard to measure hand dexterity for its excellent psychometric properties. However, caution should be taken when assessing PwMS with low (EDSS < 3) or high disability (EDSS > 6) for its floor and ceiling effects, respectively (37). We reported an overall mean score of NHPT of 0.19 pegs/s ranging between 0.23 pegs/s of the mild–moderate group to 0.12 pegs/s of the severe non-ambulant group, which is below the cutoff values previously suggested (0.27 pegs/s) (15). The comprehensive UL assessment allowed to explore UL dysfunction across neurological disability stages further. In the mild–moderate disability group, with EDSS below six, manual dexterity impairments were not associated with multiple UL dysfunctions as shown by high performances in UL function, overall UL activity, and use. Similarly, the sEMG mean level of activation was similar between patients and healthy subjects, and no significant differences were reported in the sEMG timing of activation.

Of note, sEMG alterations but not clinical dysfunctions were evident in patients with EDSS between 6 and 6.5 (severe ambulant group). The sEMG analysis suggested a progressive reduction in the modularity of muscular activation pattern according to increasing neurological disability. Modularity in muscular activation is defined as the difference between the maximum and minimum values of muscular activation (16). This dysfunction could account for the differences in the timing of maximal activation of the forearm muscles measured between the severe and the mild–moderate patients during phase 2 of the movement. In this part of the movement, patients were holding the cube and moving it to the shelf. Therefore, extensor carpi muscles were supposed to act as wrist stabilizers counterbalancing the activation of flexor muscles. The impairment of this activation timing could contribute to developing dysfunctional grasping and, as a consequence, manual dexterity deterioration. This hypothesis is supported by the significant inverse correlation measured between the NHPT and patients' abnormalities in extensor carpi timing of maximal activation compared to controls. Even in these severely impaired patients, the sEMG mean level of activation was similar to healthy subjects. Hence, sEMG data on the amplitude domain suggested that abnormalities in the timing of activation of distal UL muscles rather than the magnitude of UL muscle activation could be crucial in dexterity deterioration. This impairment could occur as a consequence of different pathophysiological mechanisms such as reduction in central drive, reduction in motoneuron excitability in the spinal cord, or reduction in sarcoplasmic reticulum calcium uptake in the skeletal muscle. Noteworthy, the only significant clinical symptom in this group was fatigue.

In the most advanced stages, with EDSS above 6.5, no further sEMG parameters deterioration were noted in distal segments. However, proximal shoulder muscles showed a delay in the maximal muscle activation in the eccentric anterior deltoid contraction during phase 3. These findings were associated with a UL disuse, impairment in grip task, and an overall decrease in UL function. Interestingly, the significant increase in fatigue observed in the previous stage (group 2) was followed by a significant decrease in the symptom's severity. A possible explanation is that the UL disuse was likely to account for lower perceived fatigue. Fatigue is considered one of the most disabling symptoms affecting PwMS, leading to the limitation in UL activities and social participation (37). Both peripheral and central mechanisms have been described in MS-related fatigue (38). In the present study, fatigue was assessed using a Numeric Rating Scale by which patients reported their overall perceived fatigue during the day while using the UL. This finding might be influenced by the nature of the assessment used and should be confirmed using more specific outcome measures of muscle endurance (i.e., handgrip or static fatigue elbow extension) (15).

So far, positive associations between manual dexterity and UL deteriorations have been reported by two clinical cross-sectional studies and one clinical instrumental observational study (5, 15, 17). The cross-sectional study by Bertoni et al. (5) explored for the first time UL dysfunctions at different ICF levels in 105 patients (5). These authors found that patients with moderate neurological disability (EDSS <4) showed limitation in manipulating small objects, while severely affected subjects with severe hand dexterity impairment showed proximal UL muscles strength deficit. Lamers et al. (15) found that different levels of hand dexterity ability based on NHPT accounted for different associations among outcome measures (15). In particular, muscle strength and active wrist mobility seemed to be more relevant in patients with severe manual dexterity impairment. Authors concluded that quantitative analysis of other factors that may contribute to UL impairment like sensorimotor function, force control, and fatigue are needed (15). A more recent study by Pellegrino et al. (17) investigated UL muscle activation pattern and coordination in different mechanical environments in 11 patients (17). The sEMG analysis showed modifications of the muscle activation pattern in PwMS compared to healthy controls during planar reaching movements in PwMS with mild–moderate UL impairment in Pellegrino et al. (17). In their study, patients were asked to perform reaching movement while grasping the handle of a robotic manipulandum. In contrast with previous findings on stroke patients (39), Pellegrino et al. (17) found no difference in the number of synergies involved in the task between patients and healthy controls. However, proximal muscles like anterior deltoid and biceps brachii showed different activation pattern compared to controls. In particular, the authors reported that shoulder muscles had different amplitude modulations and increased activity during the return phase, moving the manipulandum toward their body while flexing the elbow. Moreover, during the elbow extension, PwMS coactivated biceps and triceps brachii. Their findings suggested that the analysis of muscle activation pattern could improve the understanding of UL impairment in PwMS representing biomarkers that help in discriminating MS patients and healthy subjects.

Literature findings are partially consistent with ours. We did not find a significant association between the NHPT and Motricity Index, tremor, and sensory loss. One explanation could be the low level of tremor symptoms and a mild decrease in sensory loss present in our sample. Interestingly, a positive association was found between NHPT and UL disuse, suggesting that behavioral factors could account for severity hand dexterity impairment of PwMS. In this regard, despite the severe hand dexterity, the ARAT total score indicates a notable UL capacity in groups 1 and 2 and limited UL capacity only in group 3. UL disuse was never explored in the previous cross-sectional studies. The concept of UL disuse was derived from primary research with monkeys (6) and then extensively studied in stroke patients (7). Briefly, injury in the central nervous system (CNS) leads to sensorimotor deficits and depressed CNS. As a consequence, the patients experience fewer movements, unsuccessful motor attempts, and compensatory behavior patterns. PwMS reported 50% less use of both arms as healthy control and overall lower quality of the movement (8–11). Noteworthy, a reduction in UL use is closely related to disability and can sustain maladaptive brain reorganization (1). Specific interventions to overcome the UL disuse, however, is still under debate in PwMS (40). In the pilot randomized controlled trial by Mark et al. (40), 20 adults with hemiparetic MS were randomized to receive 35 h of either constraint-induced movement therapy or program of complementary and alternative medicine over 10 consecutive weekdays (40). Changes in the MAL was the primary outcome measure. Results suggested that constraint-induced movement therapy might increase real-world use of the more-affected arm in PwMS, and these effects might last up to 1 year. Interestingly, the training effects paralleled white matter changes.

Our findings support the use of sEMG parameters in the assessment of PwMS. Accordingly, previous work by Pellegrino et al. (16) concluded that both kinematic and electromyographic parameters might represent biomarkers that help clinicians in differentiating patients with different levels of UL motor impairment from healthy subjects (16). Surface EMG was reported to help investigate motor dysfunction as force control and fatigue in PwMS (17, 18). Results are strengthened by the sEMG protocol used during a task of reaching while grasping an object. The sEMG assessment has considerable advantages over other neurophysiological evaluation in the rehabilitation setting being portable and readily operable with different tasks (41). Considering the biology-function continuum for assessment tools in patients with CNS lesions, clinical scales inform of clinical status, providing mainly functional insight (41). Conversely, sEMG can provide an aspect of biology insight (17). Our preliminary analysis was focused on identifying changes in the modulation and timing of activation to explore the muscle coordination in functionally coupled muscles like anterior deltoid and biceps brachii, biceps and triceps brachii, flexors, and extensors carpi.

The main limitation of our study is the cross-sectional design that did not allow to track the time course of the UL deterioration and to follow the real impact of the disease on the ICF domains. Further prospective longitudinal studies should consider these limits and introduce the evaluation of all the clinical and neurophysiological data in a longitudinal prospective manner along different disease stages. In addition, the small sample allowed only a preliminary exploration of data using nonparametric tests for inferential statistic and did not allow to explore potential gender differences among groups. Cognitive assessments (i.e., attention, memory, and executive functions deficits, and mood disorders) and fatigue investigation to distinguish between central and peripheral components of the disturbance should be explored in future studies. The strengths of this study are the use of specific study population and the attempt to use a multidimensional approach to characterize UL impairments.

To conclude, the analysis of sEMG data on the amplitude domain and the association between impairments in body structure, function, activity, and participation provided new insight into the understanding of UL disability progression in PwMS. Manual dexterity should represent a primary target in PwMS rehabilitation to prevent the development of secondary UL impairment. The sEMG analysis suggests that impairments in the forearm muscle activation were associated with increasing neurological disability and UL deficits at the different ICF levels.

## Data Availability Statement

The datasets generated for this study are available on request to the corresponding author.

## Ethics Statement

The studies involving human participants were reviewed and approved by CESC prog.n.230. The patients/participants provided their written informed consent to participate in this study.

## Author Contributions

MG and NS have made substantial contributions to conception and design. AG, MCas, MCam, EC, and AP participated in the enrollment phase. JC and CD carried out the clinical assessment. NV, MF, and ED carried out instrumental assessments. SM and EB designed the algorithm for sEMG data analysis. MG and NV participated in the statistical analysis and drafted the manuscripts. NV, FF, AW, and LS participated in the manuscript revision process and gave the final approval of the version.

### Conflict of Interest

The authors declare that the research was conducted in the absence of any commercial or financial relationships that could be construed as a potential conflict of interest.
